# Protein Folding Modulates the Swapped Dimerization Mechanism of Methyl-Accepting Chemotaxis Heme Sensors

**DOI:** 10.1371/journal.pone.0046328

**Published:** 2012-09-28

**Authors:** Marta A. Silva, Tânia G. Lucas, Carlos A. Salgueiro, Cláudio M. Gomes

**Affiliations:** 1 Requimte, CQFB, Departamento de Química da Faculdade de Ciências e Tecnologia da Universidade Nova de Lisboa (FCT/UNL), Caparica, Portugal; 2 Instituto de Tecnologia Química e Biológica, Universidade Nova de Lisboa, Oeiras, Portugal; University of South Florida College of Medicine, United States of America

## Abstract

The periplasmic sensor domains GSU0582 and GSU0935 are part of methyl accepting chemotaxis proteins in the bacterium *Geobacter sulfurreducens*. Both contain one *c*-type heme group and their crystal structures revealed that these domains form swapped dimers with a PAS fold formed from the two protein chains. The swapped dimerization of these sensors is related to the mechanism of signal transduction and the formation of the swapped dimer involves significant folding changes and conformational rearrangements within each monomeric component. However, the structural changes occurring during this process are poorly understood and lack a mechanistic framework. To address this issue, we have studied the folding and stability properties of two distinct heme-sensor PAS domains, using biophysical spectroscopies. We observed substantial differences in the thermodynamic stability (ΔG = 14.6 kJ.mol^−1^ for GSU0935 and ΔG = 26.3 kJ.mol^−1^ for GSU0582), and demonstrated that the heme moiety undergoes conformational changes that match those occurring at the global protein structure. This indicates that sensing by the heme cofactor induces conformational changes that rapidly propagate to the protein structure, an effect which is directly linked to the signal transduction mechanism. Interestingly, the two analyzed proteins have distinct levels of intrinsic disorder (25% for GSU0935 and 13% for GSU0582), which correlate with conformational stability differences. This provides evidence that the sensing threshold and intensity of the propagated allosteric effect is linked to the stability of the PAS-fold, as this property modulates domain swapping and dimerization. Analysis of the PAS-domain shows that disorder segments are found either at the hinge region that controls helix motions or in connecting segments of the β-sheet interface. The latter is known to be widely involved in both intra- and intermolecular interactions, supporting the view that it's folding and stability are at the basis of the specificity and regulation of many types of PAS-containing signaling proteins.

## Introduction

Bacteria sense the chemical world by using a variety of mechanisms that couple environmental stimuli to adaptive responses. Two-component systems serve as a basic stimulus-response coupling mechanism to allow organisms to sense and respond to changes in many different environmental conditions including fundamental processes such as the regulation of gene expression, chemotaxis and signal transduction [1]–[3]. These signaling systems are characterized by a highly modular design that has been adapted and integrated into a wide variety of cellular signaling circuits [4], [5]. These signal transducers couple a regulatory heme-binding domain (sensor domain) to a neighboring transmitter (transduction domain). The more common heme-based sensors contain a *b*-type heme in the sensor domain, which is used to bind effector molecules such as O_2_, NO, or CO [6]. The binding of the effector molecule to the sensor domain induces a conformational change in the transduction domain that is responsible for the generation of the intracellular signal and subsequent regulation of the physiological function [6]–[9].

**Figure 1:**
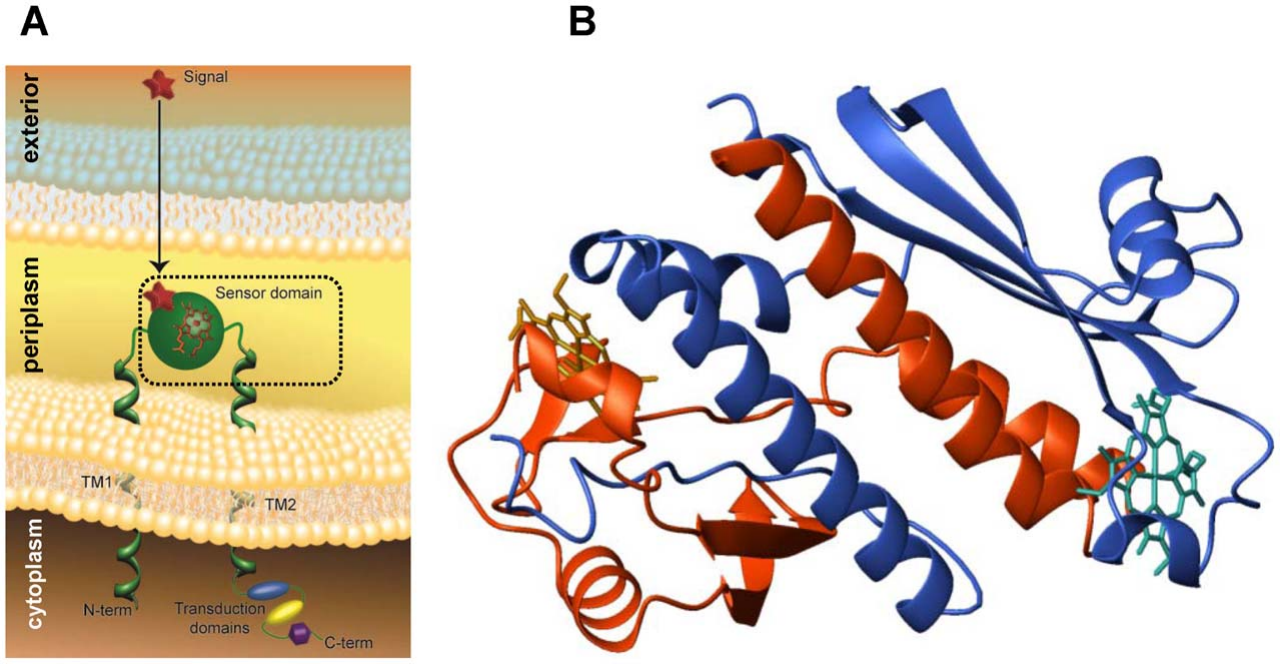
The methyl-accepting chemotaxis protein contains a periplasmic heme sensor domain. (A) Model of a methyl-accepting chemotaxis protein containing a periplasmic heme sensor domain that is anchored to the inner membrane by two transmembrane helices (TM1 and TM2). The second transmembrane helix connects the periplasmic domain to cytoplasmic transduction domains consisting of a HAMP domain followed by a methyl-accepting chemotaxis domain. (B) Structure of the helix-swapped GSU0935 sensor dimer.

*Geobacter sulfurreducens* bacterium (*Gs*) is an anaerobe microorganism that displays a considerable respiratory versatility. Thus the bacterium needs to sense quite different stimuli and this probably relates to the finding of an unusually large number of heme signal transduction proteins in its genome [10], [11]. Indeed, ten periplasmic sensor domains were found in *Gs* genome, each containing at least one heme *c*-binding motif [11], a characteristic only reported for the DcrA sensor from *Desulfovibrio vulgaris* [12], [13]. They are part of proteins annotated as two-component signal transduction or chemotaxis proteins, and have homologs in the *Geobacteraceae* family [14]. Two of the ten *c*-type heme-containing sensor domains found in *Gs*, encoded by genes *gsu0582* and *gsu093*5, are parts of chemotaxis proteins. These two sensors, hereafter designated GSU0582 and GSU0935, have similar predicted topologies: an N-terminal tail in the cytoplasm, followed by a transmembrane helix, a periplasmic domain (about 135 residues), another transmembrane helix, and cytoplasmic domains consisting of a HAMP domain followed by a methyl-accepting chemotaxis protein domain (Fig. 1A).

The crystal structures of these sensors in the oxidized form were determined and showed that they form swapped-dimers with a PAS-type fold formed by polypeptide segments of the two monomers [14]. The structures of GSU0582 and GSU0935 (Fig. 1B) heme sensors are the first representatives of a new class of heme sensors since all other heme-containing PAS sensor domains described in the literature are formed by a single polypeptide chain, located at the cytoplasm and contain *b*-type hemes [15]. The spectroscopic properties of sensor domains GSU0582 and GSU0935 in solution were also addressed by using a set of complementary spectroscopic techniques that included UV-visible, NMR, EPR and Resonance Raman [14], [16]. These studies showed that the heme group of the two sensor domains is in the high-spin form in the oxidized state and becomes low-spin after reduction. The heme groups bind nitric oxide (NO) in both the ferric and ferrous forms, whereas carbon monoxide (CO) binds only to the reduced form [14]. The measured CO and NO dissociation constants of ferrous GSU0582 and GSU0935 sensors revealed that both proteins have high and similar affinity towards these molecules but, in the ferric form, sensor GSU0582 shows a much higher affinity for NO [16]. Thus, it was suggested that the sensor domains GSU0935 and GSU0582 might use the *c*-type heme for trigger the signal transduction mechanism recognition in a process that involves the formation of the swapped dimer at periplasm with the concomitant alteration of the relative positions of the transmembrane helices and response of the transducers domains in the cytoplasm.

In this study, we set to investigate the conformation and folding properties of two distinct heme sensor PAS domains in order to determine how folding modulates dimer formation and how changes in the heme environment propagate to the global structure. Our findings suggest that differences in the thermodynamic stability and intrinsic disorder of the two sensors not only directly modulate local unfolding events that lead to swapped dimer formation, but are at the basis of distinct signaling properties and activation thresholds within heme sensor homologues.

## Materials and Methods

### Expression and purification of sensors GSU0582 and GSU0935

Expression and purification of sensors GSU0582 and GSU0935 was carried out as previously described [14]. The final yields of purified protein were 3 mg per liter of culture.

### Disorder and structural analysis

The experimental structures of sensors GSU0582 (code: 3B47) and GSU0935 (code: 3B42) were retrieved from the Protein Data Bank; the model of the GSU0935 constructed monomer as published in [14] was a kind gift from Drs. Schiffer and Pokkuluri (Argonne National Laboratory, USA). Protein structure analysis was carried out using the WhatIF web server and structural models represented using Pymol. The DisProt analysis server for intrinsically disordered protein regions (http://www.dabi.temple.edu/disprot/predictor.php) using the VL3H neural network based Predictor [17] was employed to inspect the sequences for disordered segments.

### Spectroscopic methods

Circular dichroism (CD) measurements were performed at 222 nm using a JASCO J-810 spectropolarimeter with a Peltier-thermostated cell support using a 0.1 cm path-length cell quartz. UV-visible spectra were recorded with a Shimadzu UVPC-1700 spectrometer at room temperature using a 1 cm path-length cell quartz cell.

### Analytical size-exclusion chromatography

Size-exclusion chromatography experiments were performed on an analytical-scale Superdex-75 XK-16 (V_t_ = 124 ml) column attached to Akta Prime HPLC system. The column was pre-equilibrated with 20 mM Tris-HCl pH 7.5 and 150 mM NaCl and calibrated with the following molecular mass standards: bovine serum albumin (67 kDa), ovalbumin (43 kDa), chymotrypsinogen A (25 kDa), cytochrome *c* (12.5 kDa). The partition coefficient (K_av_) was determined from equation K_av_ = (V_e_−V_o_)/(V_t_−V_o_), where V_e_ is the elution volume for the protein, V_t_ is the total bed volume and V_o_ the column void volume. Imidazole and the blue dextran 2000 were used to determine V_t_ and V_o_, respectively. In order to monitor the changes in the monomer-dimer equilibrium, sensor samples with a final concentration of 1; 2; and 3 mg.ml^−1^ were prepared and 100 µl aliquot from each sample were injected separately into the column. The runs were performed at a flow rate of 0.5 ml.min^−1^ and protein elution monitored at 280 nm.

### Equilibrium unfolding experiments

The conformational stability of the heme sensors GSU0582 and GSU0935 was assessed by performing temperature and chemical-induced denaturations, monitored by far-UV CD at 222 nm, which reports on the stability of the secondary structural elements (α-helices and β-sheets) and UV-visible absorption spectroscopy (to analyze the heme moiety). Protein solutions (0.2 mg.ml^−1^) were prepared in a 20 mM sodium phosphate buffer at different pH values covering the range 4–10. For thermal-induced denaturation, a heating rate of 1.0°C.min^−1^ was used, and temperature was increased from 10°C to 90°C. Chemical-induced denaturation experiments were carried out with several concentrations of GuHCl (in the range 0–4 M) or urea (in the range 0–9 M) at room temperature using CD and UV-visible spectroscopy. Fresh guanidinium hydrochloride (GuHCl) and urea solutions were used and their rigorous concentrations were determined from refractive index measurements. The reaction mixture was incubated for 2 h at room temperature for complete chemical denaturation. The fraction of unfolded protein (*f*_U_) was monitored by CD spectroscopy at defined denaturant concentration or temperature and calculated according to the expression *f*_U_ = (*θ_N_*−*θ*)/(*θ_N_*−*θ_U_*), with *θ_N_* being the ellipticity at 222 nm of the protein in the native folded state, *θ* the ellipticity at defined denaturant concentration or temperature, and *θ_U_* being the ellipticity at 222 nm of the completely unfolded state. The *f*_U_ was also monitored by UV-visible spectroscopy at defined denaturant concentration and calculated from the absorbance of the Soret peak at 401 nm according to the expression *f*_U_ = (*A_N_*−*A*)/(*A_N_*−*A_U_*), with *A_N_* representing the absorbance of the native state, *A* the absorbance at each intermediate concentration of denaturant, and *A_U_* the absorbance of the completely unfolded state.

### Analysis of the thermodynamic data

The GuHCl and urea unfolding transition curves for the sensors were analyzed assuming a two-state transition: N↔U, where N and U represent the native and unfolded protein, respectively. The fraction of unfolded sensor, *f_U_*, is calculated from the relationship: *f_N_*+*f_U_* = 1, where *f_N_ and f_U_* represent the protein fraction in folded and unfolded conformation, respectively. Therefore, the equilibrium constant between the unfolded and native state is given by *K* = [U]/[N] = *f_U_*/(1−*f_U_*) and is related to the standard free enthalpy of denaturation according to ΔG° = −*RT*ln*K*, where *R* is the universal gas constant (8.31 J.K^−1^.mol^−1^) and *T* is the absolute temperature. In order to estimate the conformational stability (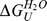) of the sensors, it was assumed that the linear dependence of the free energy of unfolding with the concentration of the denaturant continued to zero concentration [18] 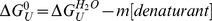, where 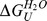 is the value of standard free enthalpy at zero concentration of denaturant (*i.e.* the conformational stability of a protein) and *m* reflects the effectiveness of the denaturant in unfolding the protein.

## Results and Discussion

### Studies of the monomer-dimer equilibrium

The heme sensor PAS domain undergoes dimerization in the methyl accepting chemotaxis protein complex. This step is believed to be crucial in the signal transduction process and the formation of the domain-swapped dimer likely involves significant folding changes and conformational rearrangements. We have thus carried out a study to warrant that at the concentrations used in the conformational stability studies, the monomer-dimer equilibrium is shifted towards the monomeric form. Indeed this was the case, as verified by using size-exclusion chromatography experiments, which showed that at working protein concentrations (typically <0.2 mg.ml^−1^) both sensor proteins eluted with estimated masses around 19 kDa, close to those calculated for the monomers (∼16 kDa) (data not shown).

### Definition of independent conformation fingerprints for heme moiety and protein conformation

**Figure 2:**
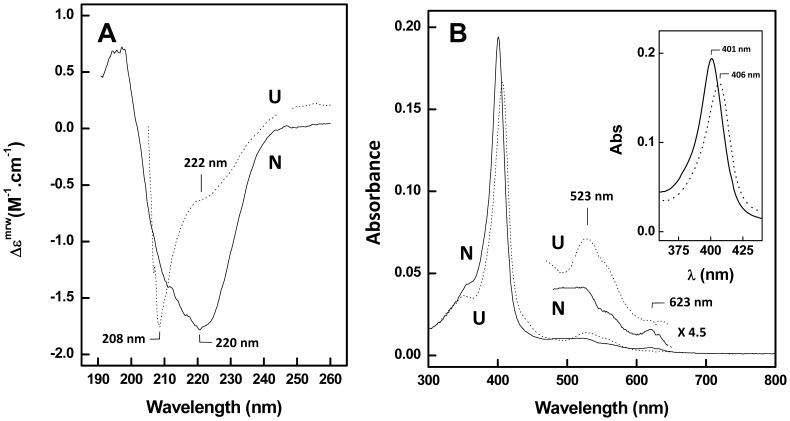
Spectroscopic characterization of GSU0935 sensor conformers. Far-UV CD (A) and UV-visible absorption (B) spectra of the GSU0935 sensor in the native (N, solid lines) and in 6 M urea unfolded state (U, dotted lines).

We then moved to establish the fingerprints corresponding to the native and unfolded conformers for the two spectroscopic methods selected for structural analysis: far-UV circular dichroism (CD) and UV-visible absorption. This combination of methodologies allows monitoring independently effects on the secondary structure (far-UV CD) and heme moiety (UV-visible absorption) during protein unfolding experiments. This will allow us to address the following questions: how do the conformational changes on the heme sensor triggered upon signaling events such as redox changes or ligand binding propagating to the PAS domain? Are structural changes within the heme environment propagating to the rest of the protein, or rather become locally restricted? This has obvious implications on the understanding of the influence of dimer interaction over the signaling mechanism. The far-UV CD spectra of the two sensor PAS proteins GSU0935 and GSU0582 are identical and can be typified by that of GSU0935 (Fig. 2A). In the native state the spectra are typical of well folded α/β proteins, featuring an intense negative band with a shoulder at 208 nm and a broader component around 222 nm (Fig. 2A, solid line). Protein unfolding results in a substantial loss of secondary structure as evidenced by a decrease in the 222 nm signal and conversion into a random coil, as reported by the emerging negative band at 208 nm (Fig. 2A, dotted line). The UV-visible spectroscopic signatures of the *c*-type heme of the two sensors allowed probing the conformational stability at the heme moiety and, thus, complementing the protein structural information obtained from the CD measurements. The electronic absorption maxima of native heme sensors is also overlapping, featuring in the oxidized form a γ-Soret band at 401 nm and weak bands in the 490–523 nm and 623 nm region (Fig. 2B, solid line). Upon unfolding of the sensors, the covalently-bound heme group remains attached to the polypeptide chain even at high concentration of denaturants, and the differences in the UV-visible spectroscopic features indicate that the heme group is a good reporter for conformational changes within the neighboring polypeptide chain. The Soret peak shifts to 406 nm and its intensity slightly decreases, whereas the 523/623 nm ratio increases (Fig. 2B, dotted line and inset). Indeed, non-covalently bound hemeproteins lose their heme group upon denaturation with a concomitant and significant shift of the Soret peak maximum to ∼365 nm [19], which is not observed in this case. We also observed that both thermal and chemical unfolding are reversible, as the far-UV CD and UV-visible absorption fingerprints corresponding to the native conformers are restored upon removal of the destabilizing condition. This allows a thermodynamic analysis of the reaction, as the system reacts under equilibrium conditions: this is not always the case among metalloproteins which frequently unfold irreversibility either due to loss or deficient reintegration of the metal cofactor upon refolding [20]–[24], although other cytochromes also undergo reversible unfolding [25]. Overall, the spectroscopic analysis of the PAS-sensor native and unfolded conformers shows that i) heme and secondary structure changes can be independently monitored as conformational probes; and ii) the reversibility of the unfolding reaction allows a quantitative thermodynamic analysis of the folding properties of both proteins. The latter is determinant for understanding mechanistic differences between the different PAS-heme sensors.

### Conformational coupling between the heme moiety and the protein fold

**Figure 3:**
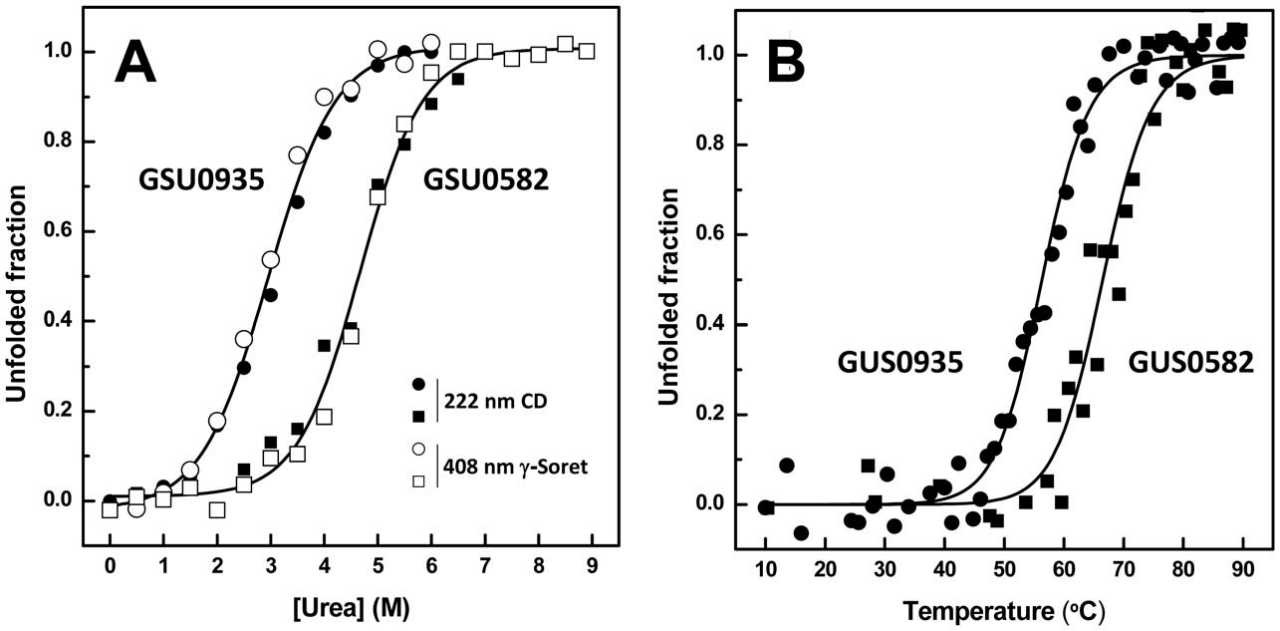
Conformational stability of the protein sensors. Chemical denaturation (A) thermal denaturation (B). The lines represent the fitting to the two-states model.

The conformational stability of PAS-heme sensors was investigated from equilibrium unfolding with the chaotropic denaturants urea and GuHCl, at pH 8. For both GSU0935 and GSU0582, the far-UV CD spectra denoted a gradual decrease in the ellipticity at increasing concentrations of denaturants, indicative of a gradual loss of secondary structure and formation of unstructured conformations. This transition was followed monitoring the variation of the CD signal at 222 nm, a wavelength at which both α-helices and β-sheets contribute significantly (Fig. 3A, closed symbols). The stability of the heme moiety was also probed upon incubation of both proteins with chemical denaturants: for both cases it is notorious that the Soret band gradually red-shifts from 401 to 406 nm, as the denaturant concentration increases (Fig. 3A, open symbols). The urea-induced unfolding curves obtained from these two complementary spectroscopic methods show that both conformational probes report the same event; in both cases, no intermediate species were observed and the denaturation curves are characterized by a sharp transition between folded and unfolded states suggesting a two-state reaction [26], [27]. This is in contrast to other heme proteins including peroxidases with unmodified heme *b*, like horseradish peroxidase or cytochrome *c* peroxidase, where unfolding is not a simple two-state process and thermodynamic intermediates are observed [19]. This suggests that the energetics of the PAS-fold GSU0935 and GSU0582 proteins is intertwined with the heme cofactor in a completely distinct way in respect to other heme-containing proteins. In fact, this coupling between heme and protein equilibrium unfolding in PAS-heme sensors is logical considering a cause-effect signaling process.

### PAS-heme sensors have distinct thermodynamic properties

Analysis of the chemical equilibrium unfolding data was carried out assuming a two-state equilibrium from which the thermodynamic parameters of the folding reactions for the two PAS-heme sensors were determined (Table 1). The same sigmoidal shape was observed for the denaturation curves obtained in presence of the denaturant GuHCl (data not shown). As expected, lower midpoint denaturant concentrations (*C_m_*) were obtained when GuHCl was used, in agreement with the fact that it is a stronger denaturant agent. Also, the cooperativity of the transitions for both sensor proteins is identical as indicated by the comparable *m* values. The slightly higher cooperativity of the unfolding transition observed for sensor GSU0582 indicates that the surface area being exposed during denaturation is higher in this protein. The occurrence of a two-state transition in both sensors reflects the covalent bonding between the prosthetic group and the protein core. The heme binds covalently to the polypeptide chain by the two cysteine residues and histidine from the heme binding motif CQSCH. This motif is located in the loop between strands β3 and β4 and is in proximity to the loop between two N-terminal helices α1 and α2 (see Fig. 1). This might explain the concerted unfolding of the heme cavity, melting of the secondary structural elements and structural disruption observed under denaturing conditions. Overall, these results indicate that the urea-mediated unfolding of both proteins is mechanistically comparable, in agreement with the fact that the two proteins have almost superimposable structures (∼1.43 Å r.m.s.). However the two proteins have striking differences in respect to their conformational stabilities, as GSU0935 sensor is substantially less stable than GSU0582 (Table 1). We have also carried out a characterization of temperature-induced unfolding followed by far-UV CD spectroscopy to monitor the thermal stability of the two proteins. For both sensors, increasing the temperature resulted in a progressive α-helix to random coil transition (Fig. 3B). At pH 8, the values of midpoint thermal unfolding, or melting temperature (*T_m_*) obtained for the two sensors are also clearly distinct: 55.8±0.2°C and 65.9±0.6°C for sensors GSU0935 and GSU0582, respectively. These values correlate extremely well with the chemical denaturation *C_m_* values, which also indicated a higher stability for sensor GSU0582.

**Table 1:** Thermodynamic parameters of denaturation of sensors GSU0935 and GSU0582 for chemical denaturation by urea and GuHCl at pH 8.

Denaturant	Parameter	GSU0935	GSU0582
**Urea**	*C_m_* (M)	3.2±0.1	4.9±0.1
	*m* (kJ.mol^−1^.M^−1^)	4.6±0.4	5.4±1.3
	_ΔG (kJ.mol_^−1^_)_	14.6±0.4	26.3±6.7
**GuHCl**	*C_m_* (M)	1.3±0.1	1.2±0.1
	*m* (kJ.mol^−1^.M^−1^)	13±5.0	16.3±5.4
	_ΔG (kJ.mol_^−1^_)_	15.9±5.0	18.8±6.7

### Electrostatic bonding contributes to differential stability of PAS-heme sensors

Although the two heme sensor domains have identical structural folds, the fact that they have different primary sequences results in the establishment of different sets of stabilizing interactions resulting in differences in conformational stability. Indeed, a number of factors influences protein stability, such as the number of hydrogen bonds, hydrophobic contacts and salts bridges, which relate to the prevalence of certain amino acid residues [28], [29]. For example, comparison of several amino acid sequences of thermophile-mesophile proteins indicate that arginine and tyrosine residues are significantly more frequent in more stable proteins, while cysteine and serine are less frequent [28]. Also, the α-helix content of glycine and alanine residues seems to contribute to protein stabilization [13]. We have carried out an analysis of amino acid sequences of the sensors under study (see below) and observed that the two proteins have rather different theoretical isoelectric points, being GSU0935 more acidic (pI∼6) than GSU0582 (pI∼7). We have thus investigated the role of electrostatic interactions on the stabilities of the two proteins by carrying out an analysis of the thermal stability as a function of pH. Using far-UV CD we are able to analyze the overall protein folding and the relative secondary structure content of the proteins when poised at distinct pH vales, as well as to determine variations in the midpoint denaturation temperature (T_m_).

**Figure 4:**
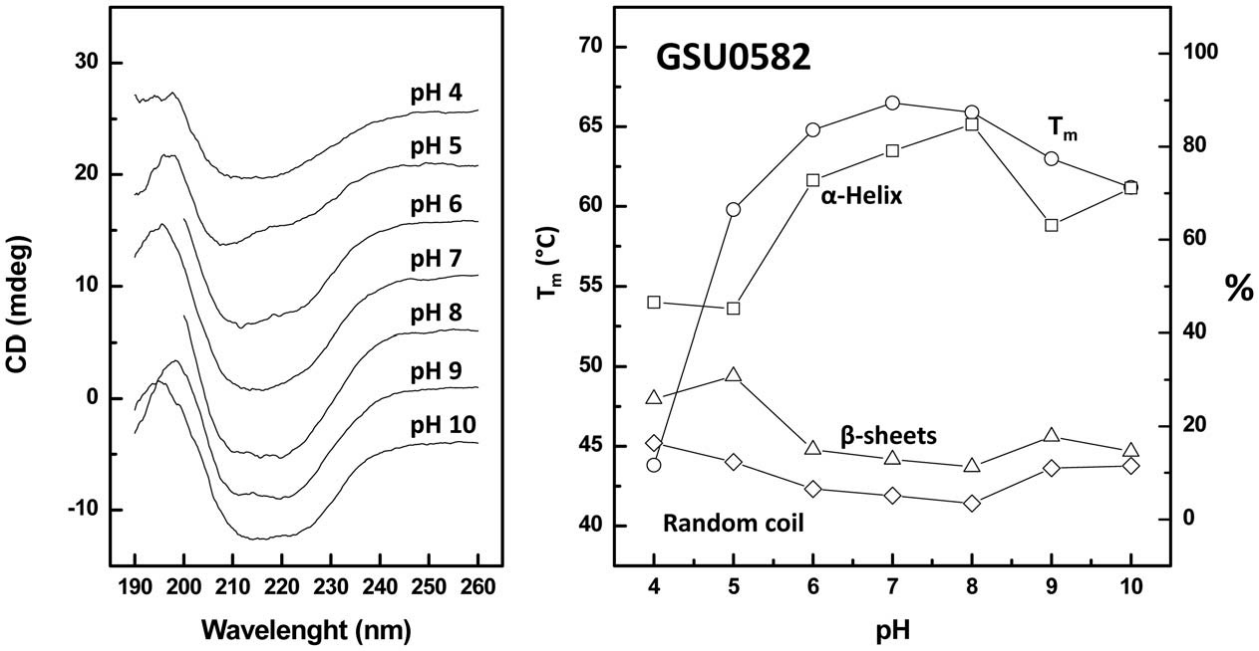
Structural (far-UV CD) and conformational characterization of GSU0582 as a function of pH. Far UV CD spectra (A) at different pH values were used to determine the melting temperature and secondary structure content as a function of pH (B).

The results obtained for sensor GSU0582 evidence minor variations in the far-UV CD spectra in the pH 6–9 range and some distortions towards the pH extremes (Fig. 4A, Table 2). These are more notorious at acidic conditions (pH<5) and in agreement this results in substantial variations in the secondary structure, with a decrease in the α-helix content and an increase in β-sheets and random structures (Fig. 4B, Table 2). Clearly, below the estimated isoelectric point side chain protonation of acidic residues results in a perturbation of the protein conformation, which is also inferred from a substantial decrease in the thermal stability from pH 6 to 4, corresponds to a ΔT_m_≈−20°C. The maximal thermal stability of GSU582 is observed between pH 6 and 8, averaging to T_m_≈66±1°C within this range. Above the estimated isoelectric point (pH 7–10), the measured T_m_ decreases only around 5°C, which suggests that, unlike acidification, increasing deprotonation and overall negative protein charges do not substantially affect the stability of the protein fold. In fact, within this range the relative variations in the secondary structure are somehow limited and the decrease in α-helix content goes along with a decrease in the T_m_: this indicates that the decreased stability of GSU0582 above pH 8 results from the fact that the protein has already a modified fold upon equilibration at higher pH, prior to thermal perturbation. These results suggest that in sensor GSU0582 a set of electrostatic interactions contributes to the maintenance of the native fold. However, in respect to other stabilizing interactions such as hydrophobic contacts and hydrogen bonding, salt bridges do not seem to contribute significantly to protein stability, as their disruption does not substantially affects the protein melting temperature.

**Table 2:** Thermal stability and secondary structure content of sensor GSU0582 as a function of pH.

pH	Thermal stability (°C)	Secondary structure (%)		
		α-helix	β-sheets	Random coil
4	43.8±0.9	47	26	16
5	59.8±0.3	45	31	12
6	64.8±0.3	73	15	7
7	66.5±0.5	79	13	5
8	65.9±0.6	85	11	3
9	63.0±0.3	63	18	11
10	61.2±0.4	71	15	12

**Figure 5:**
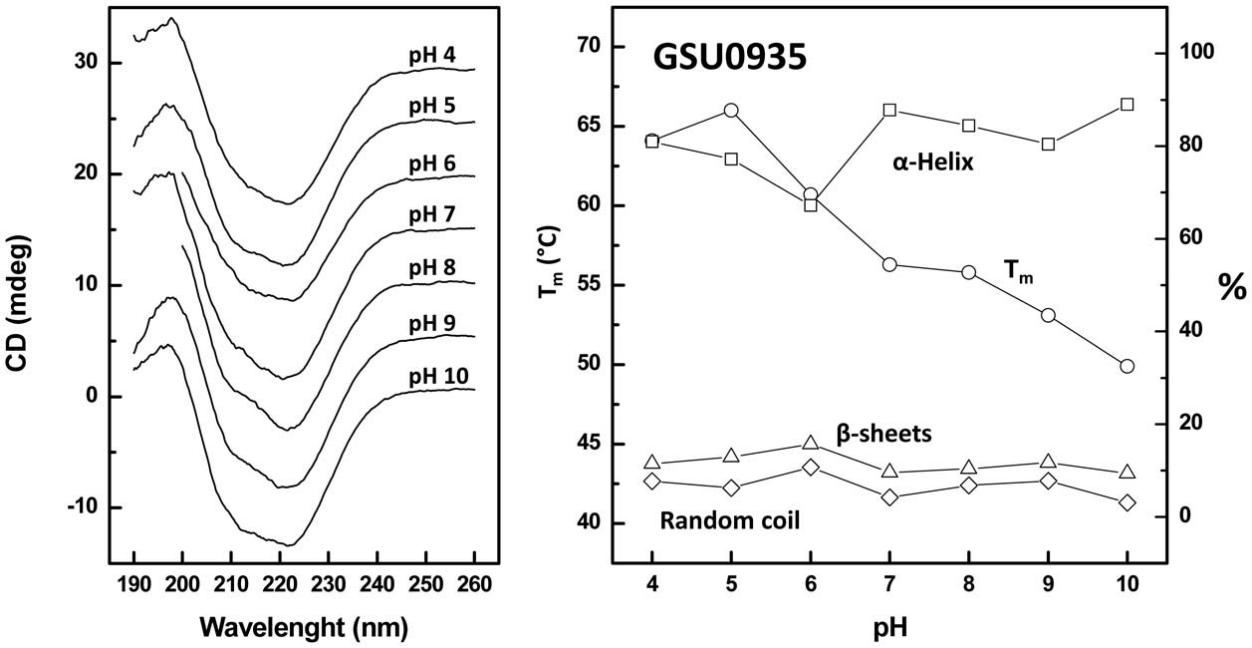
Structural (far-UV CD) and conformational characterization of GSU0935 as a function of pH. Far UV CD spectra (A) at different pH values were used to determine the melting temperature and secondary structure content as a function of pH (B).

A distinct scenario resulted from the analysis of sensor GSU0935. From pH 7 to 10 the secondary structure content is relatively invariant, as inferred from the comparable shape of the corresponding far-UV CD spectra and secondary structure quantitation (Fig. 5, Table 3). At pH 6 the α-helix content decreased but is noted to gradually recover as the protein is acidified down to pH 4, well below its theoretical isoelectric point. However, whereas the protein fold and secondary structure remain largely invariant in this broad pH range, protein stability steadily decreases with increasing pH. Contrary to what had been observed for GSU0582, these results indicate that in GSU0582 changes in protonation of charged amino acid side chains do not affect significantly the native fold but contribute significantly to protein stability, as their disruption results in a substantial decrease in the protein melting temperature (Fig. 5B, Table 3).

**Table 3:** Thermal stability and secondary structure content of sensor GSU0935 as a function of pH.

pH	Thermal stability (°C)	Secondary structure (%)		
		α-helix	β-sheets	Random coil
4	64.1±0.6	81	12	8
5	66.0±0.2	77	13	6
6	60.7±0.3	67	16	11
7	56.3±0.2	88	10	4
8	55.8±0.2	84	10	7
9	53.1±0.2	80	12	8
10	49.9±0.2	89	9	3

### Intrinsic disorder and distinct folding energetics influence swapped dimer formation

**Figure 6:**
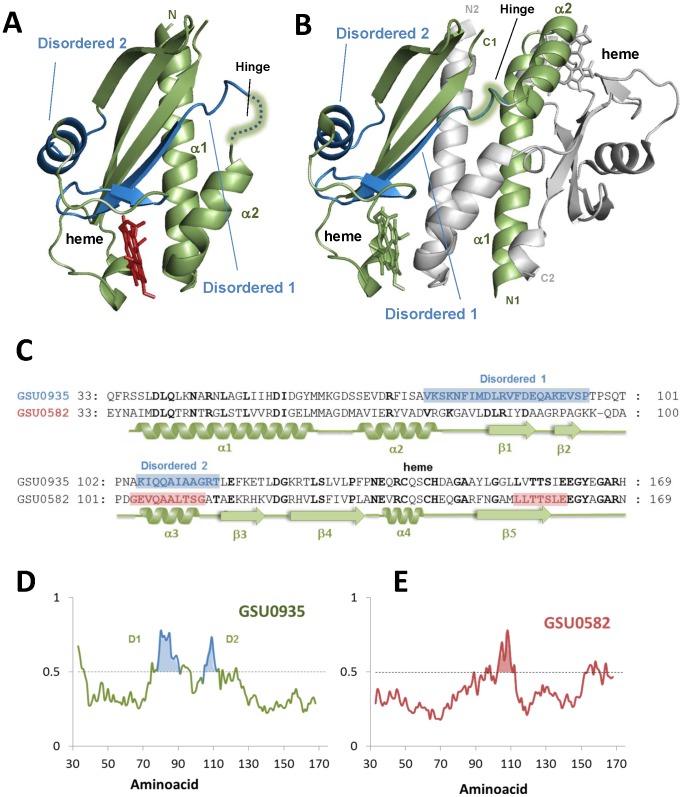
Disordered segments in the monomer and swapped dimers in PAS-heme sensors. Structure of the constructed monomer (A) and helix-swapped GSU0935 sensor dimer (B). One of the monomers is highlighted (green) and helices α1 and α2, which are displaced upon dimer assembly are specifically labeled in the figures, as well as the ‘hinge region’ through which this conformational change takes place. Analysis of the amino acid sequences of the two studied sensors (C) denotes regions of intrinsic disorder (marked in blue in the structure of GSU0935 sensor and in red in the amino acid sequence of the GSU0582). Coils and arrows denote helical (α1–α4) and strands (β1–β4) segments, respectively, and refer to the secondary structure of the crystallographic dimer. The residues that are part of the heme binding motif are bold-faced. The hinge regions in GSU0935 (…KSKNFI‥ overlaps with one of the disordered segments). The DisProt plots for intrinsic disorder prediction are shown for GSU0935 (D) and GSU0582 (E). The proteins share a disordered segment (labeled as disordered 2) in a stretch linking the five-stranded anti-parallel β-sheet region, but the thermodynamically least stable GSU0935 sensor has an additional disordered segment that covers the hinge region (labeled as disordered 1). A lower probability small disordered segment is also observed in the GSU0582 C-terminal region. N1 (C1) and N2 (C2) refer to N-terminal (C-terminal) in monomer 1 and 2, respectively.

An important aspect to address is how the conformational and stability properties of the two sensors relate to the mechanism of dimer formation. The crystal structure of sensors GSU0582 and GSU0935 show that these proteins form “swapped” dimers where the N-terminal helices from one molecule interact with the β-sheet of the other molecule (Fig. 6AB). The formation of the swapped dimer observed in the crystal structures of these two sensor domains was proposed to be a key step in the mechanism of signal transduction through the inner membrane carried out by these proteins [14]. For the swapped dimerization to occur, a significant disruption on intermolecular contacts must take place: the helical segment consisting of the two N-terminal helices (α1 and α2) of a monomer (as in Fig. 6A) has to separate from its β-sheet after a rotation of the dihedral angle in the hinge region, resulting in the swapped dimer (as in Fig. 6B) [14]. This means that this universal signaling fold must comprise some disorder: indeed this property has been observed in the PAS domain of the sensory histidine kinase Dcus, in which the disordered N-terminal helix plays an important functional role, suggesting that protein flexibility is related to the signal transduction mechanism [30]. Intrinsic disorder is a property vastly associated to signaling functions [31], and we have used the disorder predictor DisProt [17] to analyse the amino acid sequences of the two PAS-heme sensors. The results obtained showed that GSU0935 comprises 25% of disordered residues (Fig. 6C, blue amino acids), whereas in GSU0582 this number decreases to 13% (Fig. 6C, red amino acids). The plots for disorder probability (Fig. 6DE) show that both proteins share a disordered segment (labeled as disordered 2 in Fig. 6C) in a stretch linking the five-stranded anti-parallel β-sheet region, but the GSU0935 sensor has an additional disordered segment with comparable probability that covers the hinge region (labeled as disordered 1 in Fig. 6C). Since these regions are involved in domain swapping it is clear that flexibility and an intrinsic propensity to structural disorganization within these regions is an important functional property. It is then plausible that the overall stability of the PAS-domain, which determines the overall propensity to populate unfolded conformers, is strictly related to dimer formation. As expected, higher disorder and decreased conformational stability are coupled properties: the GSU0935 PAS sensor, which is the mostly disordered is also the least stable (Table 1). The thermodynamic differences observed for the two PAS-heme sensors correlate extremely well with the structural features of both sensors. The α-helix content of glycine and alanine residues in GSU0582 and GSU0935 structures is similar (Fig. 6C) and, thus, other parameters should be responsible for the different stability observed. Indeed, comparison of the amino acid sequences of GSU0582 and GSU0935 shows that the first has four more arginine and six less serine residues. The higher content of arginine residues and the smaller content of serine are certainly one of the parameters that explain the different stability of the sensors. Indeed, more stable proteins have shown higher content of residues with larger side chains, which can form salt-bridges, long range or local electrostatic and hydrophobic interactions that stabilize secondary structural elements [28]. There is also a relationship with the existence of intrinsically disordered segments: the fact that GSU0935 has a higher dependence of its stability with pH than GSU0582 likely results from its higher intrinsic disorder, as usually these proteins require a higher enthalpic stabilization to counteract their decrease in configurational entropy [32].

## Conclusion

PAS-domains are structural folds involved in signaling processes and the heme-containing PAS sensors from the bacterium *G. sulfurreducens* provide a unique framework to analyze how conformational changes effectively modulate function. In particular, the GSU0935 and GSU0582 sensors are excellent working models, as these proteins are unique examples of PAS-folded sensors undergoing swapped dimer formation. The study here reported points to the hypothesis that the primary sequence of structurally identical PAS-heme sensors dictating differences in stability will favor or disfavor the formation of the swapped dimer and thus function, as dimerization is a crucial step for signaling. Intrinsically disordered segments found within these proteins will also contribute to protein ‘fuzziness’ [32], especially as it involves linker regions that modulate conformational rearrangements, interactions and the nesting of interfaces between the two subunits. Overall, the observations here reported provide a structural and energetic framework to explain the functional properties of PAS-hemic sensors. In light of this possibility, the existence of multiple PAS-heme homologues within *G. sulfurreducens* could be related to the fact that differences in stability between otherwise structurally identical proteins result in different signaling properties and activation thresholds, thus broadening the functional range of action of the sensors.
