# Correction: Protein Folding Modulates the Swapped Dimerization Mechanism of Methyl-Accepting Chemotaxis Heme Sensors

**DOI:** 10.1371/annotation/0b01096f-01ab-4063-a15f-357dff5c0270

**Published:** 2013-03-01

**Authors:** Marta A. Silva, Tânia G. Lucas, Carlos A. Salgueiro, Cláudio M. Gomes

There was an error in the posted Funding statement. The full statement is:

This work was supported by project grants PTDC/BIA-PRO/74498/2006 (to CAS), PTDC/QUI/70101/2006 (to CMG) and by the strategic grant PEst-OE/EQB/LA0004/2011 (to the ITQB Laboratório Associado) from Fundação para a Ciência e a Tecnologia (FCT, Portugal). MAS acknowledges Fundação para a Ciência e a Tecnologia (FCT, Portugal) for the doctoral grant SFRH/BD/61952/2009. This work was also This work was also supported by the Fundação para a Ciência e a Tecnologia (Portugal)grant PEst-C/EQB/LA0006/2011 (to REQUIMTE Laboratório Associado). The funders had no role in study design, data collection and analysis, decision to publish, or preparation of the manuscript. 

